# Prefrontal, posterior parietal and sensorimotor network activity underlying speed control during walking

**DOI:** 10.3389/fnhum.2015.00247

**Published:** 2015-05-12

**Authors:** Thomas C. Bulea, Jonghyun Kim, Diane L. Damiano, Christopher J. Stanley, Hyung-Soon Park

**Affiliations:** ^1^Functional and Applied Biomechanics Section, Rehabilitation Medicine Department, National Institutes of HealthBethesda, MD, USA; ^2^Robotics Engineering Department, Daegu Gyeongbuk Institute of Science and TechnologyDaegu, South Korea; ^3^Department of Mechanical Engineering, Korea Advanced Institute of Science and TechnologyDaejeon, South Korea

**Keywords:** electroencephalography, source localization, motor cortex, gait, motor learning, gamma oscillations, event-related desynchronization, neurorehabilitation

## Abstract

Accumulating evidence suggests cortical circuits may contribute to control of human locomotion. Here, noninvasive electroencephalography (EEG) recorded from able-bodied volunteers during a novel treadmill walking paradigm was used to assess neural correlates of walking. A systematic processing method, including a recently developed subspace reconstruction algorithm, reduced movement-related EEG artifact prior to independent component analysis and dipole source localization. We quantified cortical activity while participants tracked slow and fast target speeds across two treadmill conditions: an active mode that adjusted belt speed based on user movements and a passive mode reflecting a typical treadmill. Our results reveal frequency specific, multi-focal task related changes in cortical oscillations elicited by active walking. Low γ band power, localized to the prefrontal and posterior parietal cortices, was significantly increased during double support and early swing phases, critical points in the gait cycle since the active controller adjusted speed based on pelvis position and swing foot velocity. These phasic γ band synchronizations provide evidence that prefrontal and posterior parietal networks, previously implicated in visuo-spatial and somotosensory integration, are engaged to enhance lower limb control during gait. Sustained μ and β band desynchronization within sensorimotor cortex, a neural correlate for movement, was observed during walking thereby validating our methods for isolating cortical activity. Our results also demonstrate the utility of EEG recorded during locomotion for probing the multi-regional cortical networks which underpin its execution. For example, the cortical network engagement elicited by the active treadmill suggests that it may enhance neuroplasticity for more effective motor training.

## Introduction

There is mounting evidence of cortical involvement in walking facilitated by the advancement of mobile functional neuroimaging technologies, namely functional near infrared spectroscopy (fNIRS) and electroencephalography (EEG), which have allowed for the study of brain activation during gait. fNIRS uses infrared light to assess changes in oxygenated (HbO) and deoxygenated (HbR) hemoglobin levels in the outer layers of the cortex (Boas et al., 2001). Increased HbO levels in the primary motor cortex and supplementary motor area during walking have been reported (Miyai et al., 2001). Another study found elevated HbO levels in the prefrontal, premotor, and medial sensorimotor cortices during the transition from standing to walking, however, HbO levels returned to baseline or below once a steady walking speed was reached (Suzuki et al., 2004). Interestingly, the same study showed that the increase in prefrontal and premotor HbO was greater for faster gait speeds, while the sensorimotor cortex activation was not related to gait speed. In agreement with these results, another study found increased activation of the prefrontal cortex and supplementary motor area before walking and precision stepping while primary motor and somatosensory cortices showed increased activity at gait initiation but then quickly returned to baseline levels for both tasks (Koenraadt et al., 2014). Increased gait variability and complexity also appear to increase cortical activity in the motor cortex and supplementary motor area (Kurz et al., 2012).

Similar to fMRI, fNIRS has limited temporal resolution due to the slow nature of hemodynamics. It is therefore unable to identify intra-stride patterns of cortical activity during gait. EEG is particularly well suited to monitor changes in cortical activity during locomotion due to its unencumbering wireless sensors, relatively dense scalp coverage, and high temporal resolution, which allows examination of gait-related changes in brain activation patterns. One of the first published studies of EEG during walking utilized independent component analysis (ICA) and equivalent dipole fitting to reveal intra-stride changes in spectral power coupled to the gait cycle in anterior cingulate, posterior parietal, and sensorimotor cortices (Gwin et al., 2011). Another study found similar gait-related patterns of spectral power in EEG electrodes over the motor cortex, as well as event related desynchronization (ERD), or a decrease in spectral power, in the μ (8–12 Hz) band in the medial motor cortex and in the β (18–26 Hz) band over the lateral motor cortices during treadmill walking compared to standing (Severens et al., 2012). ERD is a well established correlate of movement related cortical activity (Pfurtscheller and Lopes da Silva, 1999). Additionally, delta band EEG has been used to reconstruct lower extremity kinematics during treadmill walking (Presacco et al., 2011, 2012). A significant level of coherence between EEG recorded over the medial sensorimotor cortex and tibialis anterior EMG was observed in lower γ frequency bands (24–40 Hz) during walking (Petersen et al., 2012). High density EEG has also been used to study effective connectivity between different cortical areas during locomotion, with one study showing decreased connectivity between areas involving sensorimotor cortices during walking compared to standing, while connectivity between non-sensorimotor areas was stronger during gait (Lau et al., 2014).

Recent studies of robot assisted stepping and body-weight supported treadmill walking have also implicated cortical involvement in gait. An fNIRS study found decreased activity in the sensorimotor cortex of stroke survivors while they walked with body weight support that improved gait asymmetry (Miyai et al., 2006). One study of healthy individuals observed μ and β ERD over the central midline of the motor cortex during robot assisted stepping (Wieser et al., 2010). Enhanced μ and β ERD was reported when subjects attempted to actively walk with a robotic gait trainer as opposed to passively allowing it to move their legs (Wagner et al., 2012). Furthermore, sustained ERD was present in sensorimotor cortices for the upper μ and β frequency bands during walking with a robotic gait orthosis (Seeber et al., 2014). Spectral power in the μ and β bands was suppressed in clusters of independent components from the pre-motor and posterior parietal cortex, areas linked to motor planning, during robot assisted treadmill walking in an interactive virtual environment compared to a traditional setup with no visual feedback (Wagner et al., 2014). Cortical activation patterns at higher frequencies have also been linked to gait. Low γ band (25–40 Hz) desynchronization and synchronization modulated according to gait cycle phase was observed in the premotor cortex (Wagner et al., 2012). Similar low γ modulations localized to the central sensorimotor cortex were reported in another study (Seeber et al., 2014). These patterns may be task dependent since modulations of low γ band power in the pre-motor cortex were decreased during walking in a virtual environment (Wagner et al., 2014).

A chief purported advantage of body weight supported and robot assisted gait training is the repeatable nature of the movement which was hypothesized to induce task-dependent neuroplasticity (Dobkin, 1999). The slow walking speeds and relatively static nature of the locomotion also limit artifacts present in EEG collected during these training tasks. However, increasing evidence from randomized controlled trials indicates that treadmill based training paradigms do not produce superior outcomes compared to traditional therapies (Brown et al., 2005; Hidler et al., 2009; Willoughby et al., 2010; Duncan et al., 2011; Dobkin and Duncan, 2012; Vaney et al., 2012). Recent studies implicating active participation and individual motivation as crucial elements of neural plasticity for motor memory (Lotze et al., 2003; Beck et al., 2007) and evidence demonstrating activation of distributed cortical networks during motor learning (Dayan and Cohen, 2011) may explain the lack of motor skill acquisition from these treadmill training approaches that involve walking—or allowing a robot to move the limbs—at a constant pace on a flat unchanging surface since this task requires minimal mental engagement. Treadmill walking also differs biomechanically from overground walking (Alton et al., 1998; Dingwell et al., 2001; Kautz et al., 2011) and thus the ability to translate locomotor skills acquired in this setting may be reduced, as is the ability to decipher the role supraspinal circuits in control of natural locomotion from EEG recorded in it. As a solution to these challenges, we developed a user-driven treadmill control scheme that simulates overground locomotion (Kim et al., 2012, in press; Yoon et al., 2012). This active treadmill uses a motion-capture based controller to automatically adapt treadmill belt speed to user gait speed during each step, while compensating for the anomalous inertial force due to belt acceleration, thus providing for natural changes in speed similar to overground walking. A previous study found no differences in cadence, step length, or pelvic acceleration between a typical treadmill and the active treadmill which estimated walking speed based on pelvis and swing foot velocity (Yoon et al., 2012).

As discussed above, inclusion of EEG in gait training paradigms has the potential to characterize cortical dynamics underlying movement execution while also providing a method to gauge attention to the task. However, an inherent challenge of EEG collected during ambulation is the likely addition of gait-related movement artifacts to typical sources of noise already present in EEG signals. A recent study observed similar power fluctuations during treadmill walking from EEG electrodes and an accelerometer mounted on the head (Castermans et al., 2014). The study focused on intra-stride changes in spectral power and did not examine ERD in the μ and β bands. Nevertheless, gait-related artifact in the EEG appears to be present at a wide range of frequencies and thus, a sophisticated strategy for removing these artifacts is critical for EEG recorded during locomotion. Spatial filtering techniques, such as independent component analysis (Delorme et al., 2007) or canonical correlation analysis (De Clercq et al., 2006), have been previously applied to remove artifacts from EEG. While effective at identifying spatially and spectrally distinct sources of artifact, such as EMG, EOG, or EKG, these blind source separation techniques may have difficulty fully parsing movement artifacts present in EEG recorded during gait. As a result, one study developed a template regression technique to clean EEG of all signals coupled to the gait cycle (Gwin et al., 2010); however, this type of procedure may not be ideal for studying the cortical contribution to locomotion because meaningful brain activity could be removed along with the artifact. Another recently developed algorithm uses principal component analysis to compare sliding windows of EEG to a baseline recording, thereby removing non-stationary, high variance (high amplitude) artifacts and reconstructing the removed sections using neighboring channels and a mixing matrix based on the calibration data (Mullen et al., 2013). To our knowledge, this approach has not previously been applied to EEG collected during gait.

In this study a systematic EEG data analysis strategy, designed to reduce motion artifact while minimizing loss of useful data, was used to examine cortical activation patterns of able-bodied adults during simulated overground walking with our novel user-driven (active) treadmill and walking with a typical (passive) treadmill. We hypothesized that active control would increase cortical activity and voluntary drive compared to passive treadmill walking regardless of gait speed; a result that would elucidate supraspinal networks involved in limb coordination during walking and provide impetus to include such control in treadmill based rehabilitation.

## Materials and methods

### Experimental protocol and data collection

Ten healthy adults (6 female, 4 male; age: 28.9 ± 6.3 years; height: 165 ± 9 cm; weight: 65.6 ± 12.5 kg) with no history of neurological disease participated in this study. The experimental protocol was approved by the institutional review board of the National Institutes of Health. All subjects gave written consent prior to study participation. While all 10 subjects completed the protocol, a technical problem during data collection resulted in unusable data from one subject; thus the data analysis was performed on data from the nine remaining subjects. Prior to the experiment, each subject was fitted with a 64-channel, wireless, active electrode EEG system (Brain Products, Morrisville, NC) with sensors placed according to the 5% 10–20 international system (Easy Cap, Germany) with the reference placed at the FCz electrode location. Electrode impedance was maintained below 20 kΩ. EEG data were sampled at 500 Hz. Reflective markers placed on the right and left posterior superior iliac spines (PSIS) and on the right and left feet were tracked using a Vicon MX motion capture system (Vicon, Denver, CO) at 120 Hz.

Participants walked at two command speeds: slow (0.9 m/s) and fast (1.5 m/s). Prior to walking, we assessed user comfort with each speed and for one subject gait speeds were adjusted down to 0.8 and 1.4 m/s. Each participant completed a total of six walking trials on a split belt treadmill (Bertec Co., Columbus, OH), three each in two different modes: typical (passive) and user-driven (active) walking. The passive walking mode reflected a typical treadmill: belt speed was automatically adjusted to the target speed within each block (slow or fast) so that walking speed was dictated by the treadmill setting. In the active mode a combination of feedforward and feedback controllers were implemented to allow the user to drive the speed of the treadmill. Briefly, the desired gait speed of the user was estimated from the maximum foot swing velocity in the sagittal plane (Yoon et al., 2012), which allows for adaptation of the belt speed within the first half of a step. A smart estimation limiter adjusted the acceleration of the belt during speed adaptation to maintain similar ground reaction forces as those experienced during overground walking (Kim et al., in press).

**Figure 1:**
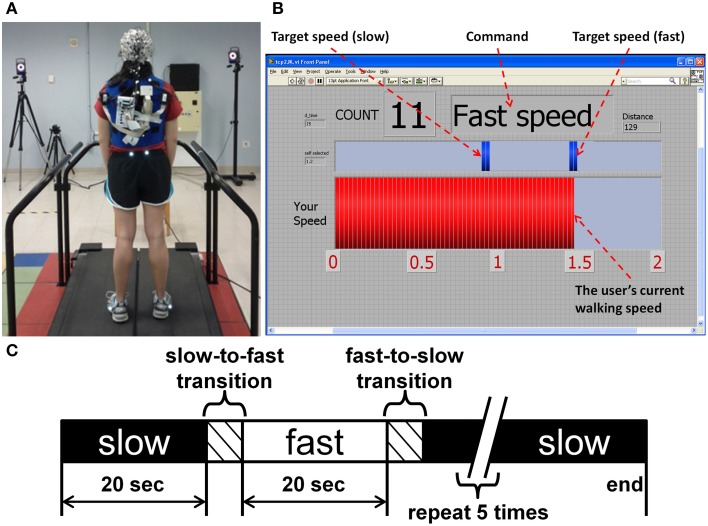
**(A)** The experimental setup for EEG data collection during treadmill walking. **(B)** Computer screen providing real-time feedback of speed performance to the participant. **(C)** Block design of each trial.

Each trial consisted of ten 20 s blocks of walking alternating between slow and fast command speeds (Figure 1). A monitor placed in front of the treadmill displayed the target walking speed and the user's current walking speed as measured by the motion capture system. Within each block, the participant was instructed to match and maintain their walking speed to the command speed (slow or fast). Prior to data collection each participant practiced walking with the passive and active modes for 5 min. Finally, 1 min of EEG was collected during quiet standing before and after all walking trials were completed.

### Signal processing

All data analyses were performed off-line using custom software in Matlab (Mathworks, Natick MA) containing functions from EEGLAB v13 (Delorme and Makeig, 2004). Gait events (heel strike and toe-off) were determined from the foot markers and treadmill force plate data. Pelvis velocity was computed by differentiating the trajectory of the pelvis from the motion capture data while belt velocity was computed by differentiating the foot marker position when it was in contact with the belt during each step. Walking speed reported to the user (Figure 1B, red bar) was computed in real time as belt velocity plus the pelvis velocity in the sagittal plane.

**Figure 2:**
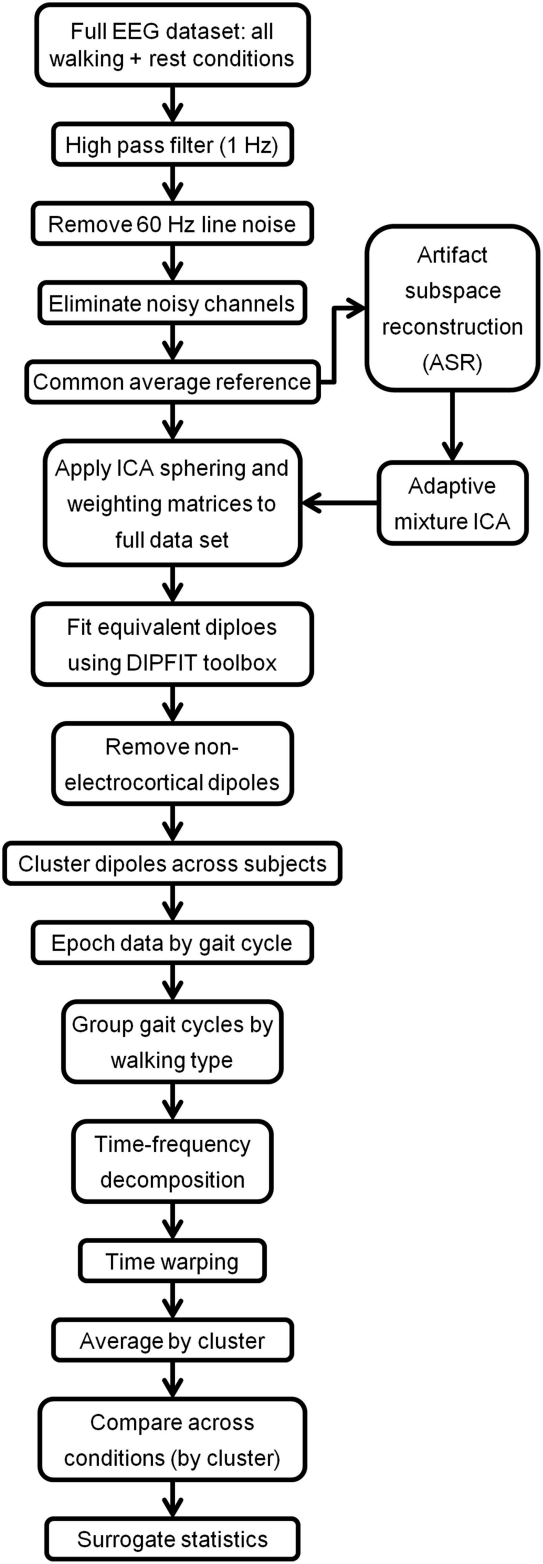
**Flow-chart indicating the EEG processing pipeline for treadmill walking**.

The EEG signal processing methodology is shown in Figure 2. For each subject, all walking trials and the rest (quiet standing) trials were concatenated into a single EEG dataset. The EEG signals were high pass filtered at 1 Hz (5th order Butterworth) and time-locked to the kinematics. Power line noise at 60 and 120 Hz was removed using the cleanline function from EEGLAB. Noisy EEG channels, indicated by a standard deviation greater than 1000 μV or a kurtosis of more than 5 standard deviations from the mean were removed (Gwin et al., 2011). An average of 63 channels (range: 61–64) were retained per subject. The EEG was then re-referenced to a common average of the remaining channels. Next, an artifact subspace reconstruction (ASR) algorithm adapted from EEGLAB software (Mullen et al., 2013) was implemented to remove high amplitude artifact from the EEG recorded during walking. ASR transforms a sliding window of EEG data with principal component analysis to identify channels of high variance by statistical comparison with clean EEG data containing minimal movement artifact. Using the principle of volume conduction, single channels capturing true electrocortical signals should not principally account for a large amount of variance within the given window. Channels which show variance above a threshold compared to calibration data are identified as corrupted. Here, 2 min of EEG recorded during quiet standing was used as calibration data for ASR. Corrupted channels (or subspaces of multiple channels) were reconstructed from neighboring channels using a mixing matrix that is computed from the covariance matrix of the calibration data, again based on the principle of volume conduction. In this study a sliding window of 500 ms and a variance threshold of three standard deviations were used to identify corrupted subspaces.

After ASR, an adaptive mixture independent component analysis (AMICA) (Palmer et al., 2008) technique was applied to the cleaned EEG data from each subject to separate the EEG recordings, a combination of individual brain and non-brain sources mixed by volume conduction, into spatially static independent components (ICs) of maximal temporal independence. While many blind source separation algorithms exist, AMICA has been shown to maximize mutual information reduction and the dipolarity of scalp projections following decomposition (Delorme et al., 2012). AMICA was performed on 22 min of EEG data for each subject. Thus, the number of time points used to estimate the weighting matrix ranged from 161–177 times the number of channels squared, exceeding the recommended amount for satisfactory decomposition (Delorme and Makeig, 2004). To minimize any possible loss of electrocortical signals from ASR, sphering and weighting matrices obtained from AMICA decomposition of the cleaned data set were applied to the full preprocessed EEG data set (Figure 2). Next, an equivalent dipole current source was fit to each IC from a three shell boundary element model using the DIPFIT toolbox of EEGLAB (Oostenveld and Oostendorp, 2002). The EEG electrode positions were aligned to fit the standard MNI brain template (Montreal Neurological Institute, Quebec, Canada). Only dipoles that accounted for at least 80% of the variance for a given IC scalp projection were retained for further analysis. The remaining dipoles for each subject were categorized as a brain or non-brain source based on the dipole location (Talairach coordinates), power spectra, and time traces of voltage. On average, we identified 7 (range: 4–10) brain dipoles for each subject. These ICs were then clustered across subjects using feature vectors formulated with dimensions for power spectral density (<100 Hz) and dipole location (Gwin et al., 2010; Wagner et al., 2012). Using EEGLAB functions, feature vectors were reduced to 10 principal components and clustered across subjects using *k*-means (*k* = 7). ICs greater than three standard deviations from a cluster centroid were relegated to an outlier cluster and subsequently omitted from analysis.

Each IC was then split into 2.2 s epochs surrounding the right heel-strike (RHS), starting from 200 ms before each RHS. Each epoch was labeled according to its corresponding walking command: slow, fast, slow-to-fast transition (acceleration), or fast-to-slow transition (deceleration) and treadmill type: passive or active. Transitions were identified as the six gait cycles—three before and three after—surrounding the change in command speed, a number that assured all subjects had reached steady state based on *post-hoc* analysis of gait speed. Next, epochs labeled as acceleration and deceleration were removed from the data set so that only gait cycles containing steady slow and fast walking were retained for analysis, resulting in four experimental conditions: slow passive, slow active, fast passive, and fast active. We computed the power spectral density (PSD) for each epoch with a multitaper fast Fourier transform with discrete prolate sequences using the “*pmtm”* command in Matlab. The PSD was then averaged across all epochs for each IC for a given experimental condition. Cluster grand mean PSDs were computed by averaging across the ICs within each one. A within subjects, repeated measures Two-Way ANOVA with Bonferroni correction for multiple comparisons was used to assess significant effects of speed (two levels: slow, fast) and treadmill type (two levels: passive, active) on area under the PSD curve of each cluster across five frequency bands of interest: Δ (1–4 Hz), θ (4–8 Hz), α/μ (8–13 Hz), β (14–30 Hz), and low γ (30–50 Hz). Shapiro-Wilk tests of normality on studentized residuals verified that PSD data within all bands and clusters were normally distributed.

The time-frequency decomposition (spectrogram) of each epoch was computed using a fast Fourier transform with a Hanning window taper. Within each cluster, single epoch spectrograms from each IC were linearly time-warped so that gait events (heel-strikes and toe-offs) occurred at the same median latency (Gwin et al., 2011). To elucidate intra-stride modulations of power for each IC, we computed logarithmic time-frequency magnitude ratios via division of each time point in the spectrogram by the gait cycle mean log spectrum for the respective experimental condition, resulting in time-frequency plots showing spectral power changes relative to the average activity over the gait cycle. These event-related spectral perturbations (ERSPs) were averaged across all ICs in a cluster to create grand mean ERSPs for each walking condition. Significant ERSP values (*p* < 0.05) were identified using a bootstrapping technique within EEGLAB (Delorme and Makeig, 2004).

Quiet standing data from each IC was split into 2.2 s epochs and spectrograms were computed to serve as a baseline. Individual epoch spectrograms were averaged for each IC from the four walking conditions and the baseline (quiet standing) condition. The mean baseline condition was then subtracted from the four walking conditions. Finally, these mean baseline subtracted spectrograms were averaged across all ICs in a cluster for each experimental condition. To ascertain significant differences between active and passive treadmill we transformed the time-frequency values to decibels (dB) and statistically compared spectrograms using a non-parametric bootstrapping technique with random shuffling (Delorme and Makeig, 2004). We generated 2000 surrogate data sets and significant differences were those outside the 95% confidence interval (α < 0.05). To visualize the difference between treadmill types at each speed, we computed difference spectrograms by subtracting the two relevant conditions (e.g., slow passive walking from slow active walking). In addition to providing a measure of ERD/ERS over the course of the gait cycle, this subtraction procedure also mitigates the effect of any residual artifacts when treadmill conditions (passive and active) are compared since the kinematics between the two are similar (Yoon et al., 2012; Kim et al., in press). These difference spectrograms were masked for significance using the surrogate statistics.

## Results

**Figure 3:**
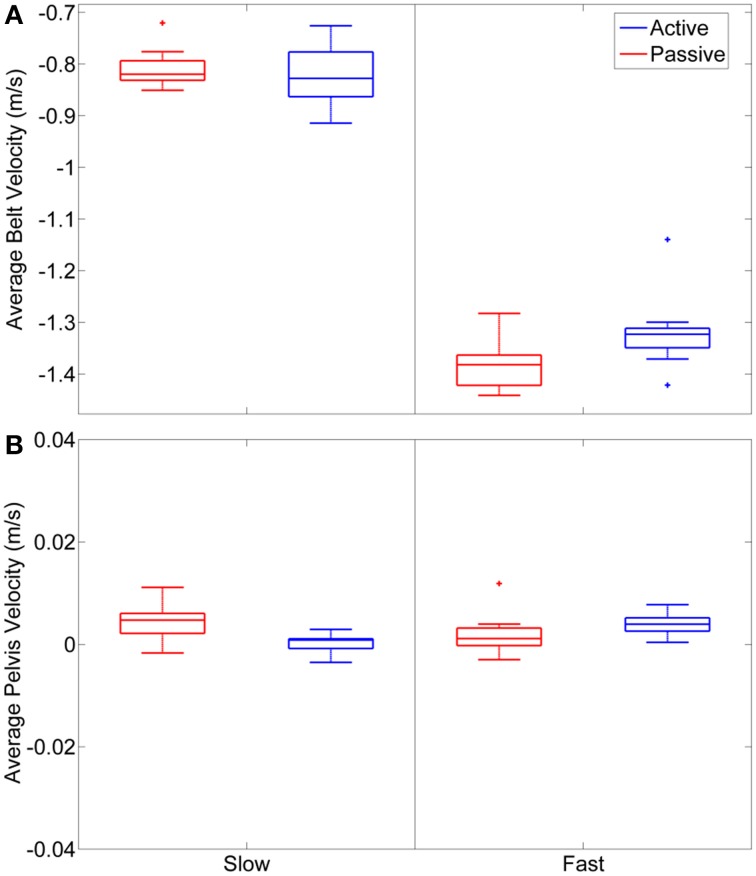
**(A)** Average belt velocity, measured as foot velocity during stance phase, for slow and fast walking commands in passive and active mode across the 9 subjects. **(B)** Average sagittal plane pelvis velocity during the treadmill walking tasks.

As expected the average treadmill belt velocity was more variable during active treadmill walking, regardless of command speed (Figure 3A). Average pelvis velocity in the sagittal plane was similar for the slow and fast walking commands, indicating the participants were able to match the desired treadmill command speed accurately during the steady walking tasks (Figure 3B). The *k*-means clustering resulted in seven spatially distinct clusters of electrocortical dipole sources, plus one additional outlier cluster (Table 1). We identified the Brodmann areas represented within each cluster from the Talairach atlas (Lancaster et al., 2000); dipoles were located within ±3 mm cube range of 14 Brodmann areas across the seven clusters. We performed spectral and time-frequency analysis on 6/7 clusters with the RPM cluster omitted because it did not contain ICs from a majority of the participants.

**Table 1:** **Clusters of dipolar sources fit to independent components**.

	**Posterior parietal**	**Left motor**	**Right motor**	**Left premotor**	**Right premotor^a^**	**Anterior cingulate**	**Prefrontal**
	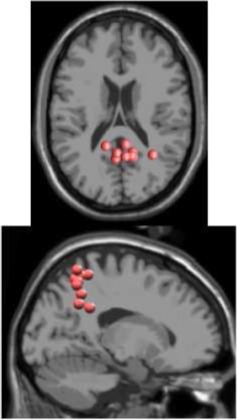	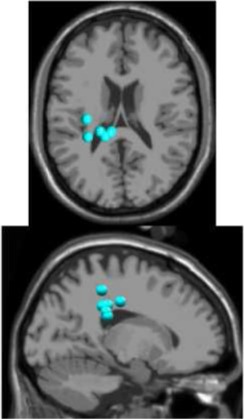	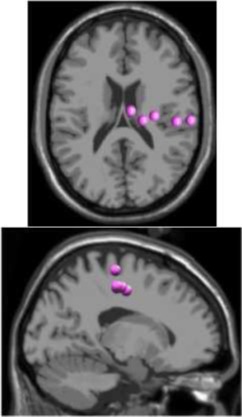	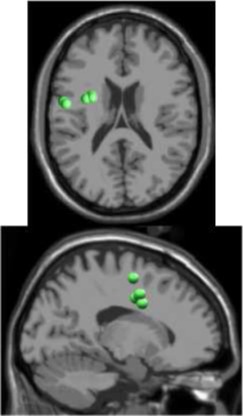	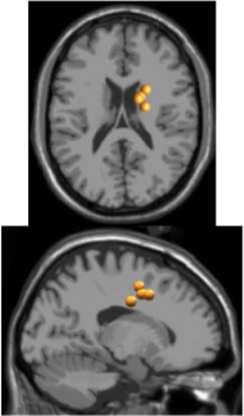	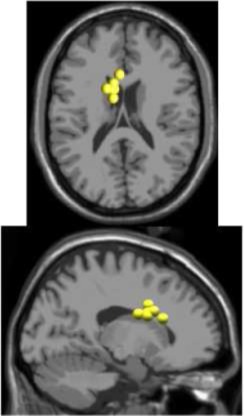	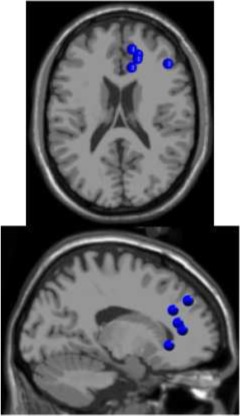
Number of subjects	7	5	5	5	3	5	5
Number of ICs	9	5	5	5	4	5	5
Brodmann areas^b^	5, 7, 31	3, 4, 31	1, 3, 4, 6	6, 8, 24	6, 24	24, 32, 33	9, 10, 32, 46

*^a^The RPM cluster was omitted from analysis because it did not contain ICs from a majority of the subjects*.

*^b^Brodmann Areas reflect those found within a ±3 mm search range of all individual dipoles within a cluster*.

**Figure 4:**
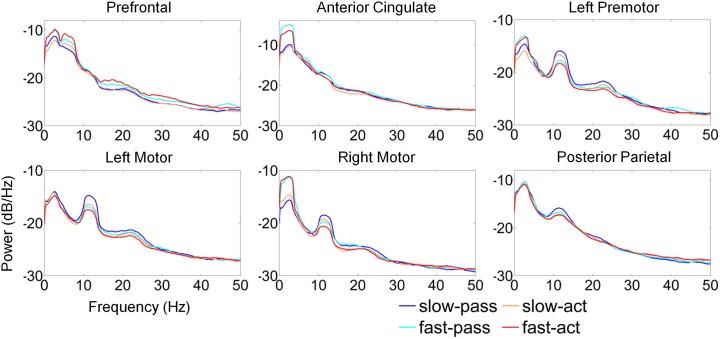
**Average power spectra by cluster for the four experimental walking conditions**. (1) Passive treadmill, slow speed (slow-pass); (2) passive treadmill, fast speed (fast-pass); (3) active treadmill, slow speed (slow-act); and (4) active treadmill, fast speed (fast-act).

There was a significant interaction effect between speed and treadmill conditions on μ band PSD in the left motor (LM), right motor (RM), and posterior parietal (PP) cortex (Table 2). *Post-hoc* tests revealed a significant decrease in μ band power in LM and RM during active compared to passive treadmill walking at slow speed, and at both slow and fast speeds in PP (Figure 4, Table 2). *Post-hoc* tests also showed significantly decreased μ band power for fast compared to slow walking in the LM for both passive and active modes and for passive mode in PP. β band power in the RM cluster was significantly decreased [*F*_(1, 4)_ = 61.0; *p* < 0.01] for active compared to passive treadmill walking (Figure 4). The only significant interaction for β band was in the PP region, with significant power decrease for active vs. passive treadmill at both speeds and for fast vs. slow walking in both treadmill modes (Table 2). Two main effects were observed for θ band power, one in the PP region for treadmill type [*F*_(1, 8)_ = 19.4; *p* = 0.01] with less power for active compared to passive walking and one in the prefrontal (PF) region for speed [*F*_(1, 4)_ = 30.1; *p* = 0.04] with increased power for fast compared to slow walking. The only significance for γ band PSD was a main effect for treadmill type in the PP cluster [*F*_(1, 8)_ = 12.2; *p* = 0.04] with power increased for active compared to passive mode. There were no significant interactions or main effects on PSD for treadmill type or speed for the anterior cingulate (AC) or left premotor (LPM) regions in any frequency band.

**Table 2:** **Significant interaction effects between speed and treadmill type on spectral power**.

	**μ Band**					**β Band**				
**Region**		**Effect of speed**		**Effect of treadmill**			**Effect of speed**		**Effect of treadmill**	
	**Interaction**	**Passive**	**Active**	**Slow**	**Fast**	**Interaction**	**Passive**	**Active**	**Slow**	**Fast**
LM^a^	*F*_(1, 4)_ = 14.5; *p* = 0.03	*p* < 0.01	*p* = 0.04	*p* = 0.02	ns	ns				
RM^a^	*F*_(1, 4)_ = 13.0; *p* = 0.02	ns	ns	*p* = 0.01	ns	ns				
PP	*F*_(1, 8)_ = 18.9; *p* = 0.01	*p* < 0.01	ns	*p* < 0.01	*p* < 0.01	*F*_(1, 8)_ = 13.4; *p* = 0.03	*p* < 0.01	*p* = 0.01	*p* < 0.01	*p* < 0.01

*^a^No significant interaction was observed in LM or RM for β band*.

**Figure 5:**
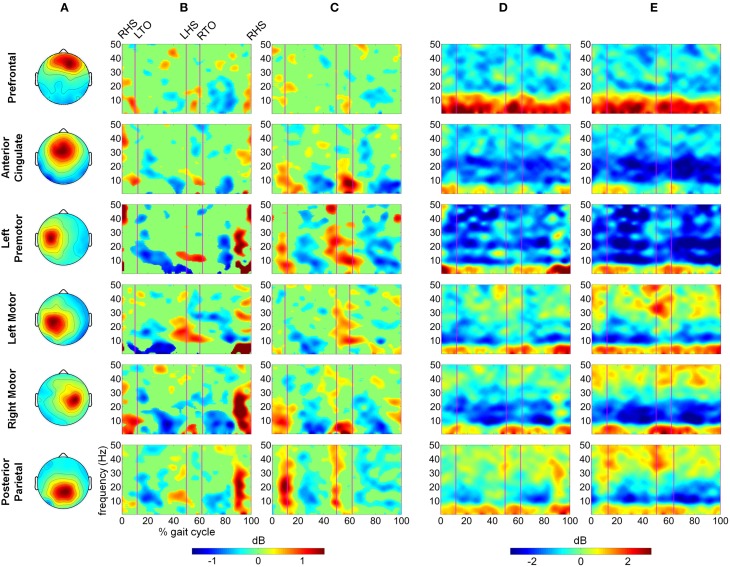
**Time frequency decomposition of cortical clusters during steady, slow treadmill walking. (A)** The average topographical scalp projections of independent components comprising the six cortical clusters. Grand mean time frequency amplitude ratio to the mean gait cycle spectrum, constituting gait cycle event related spectral perturbations (ERSPs) for each cluster during **(B)** passive and **(C)** active treadmill walking. Event related desynchronization (ERD) and synchronization (ERS) during **(D)** passive and **(E)** active treadmill walking relative to quiet standing for each cluster.

Amplitude modulations relative to the mean gait cycle activity were present in the μ and lower β band of LM, RM, and PP clusters with similar temporal patterns (Figures 5B,C). ERSPs showing activity up to 100 Hz can be found in Supplementary Figure 1. The PP modulations were broad, extending through the β and γ frequency bands. In the RM cluster γ band modulations were shifted relative to μ and β rhythms, with γ desynchronization during early swing phases and synchronization during late swing. The type of treadmill (active or passive) did not affect gait cycle related modulations in the LM, RM, or PP. However, in AC and LPM, gait-cycle related μ band modulations were stronger during active treadmill walking. Furthermore, β and low γ band modulations were present in these clusters during active walking.

The time-frequency analysis showed changes in spectral power at multiple frequency bands during steady walking compared to quiet standing (Figure 5). We observed sustained ERD in the β band across all 6 clusters and in the μ band for all clusters except for PF. ERS was observed in low frequency bands (Δ and θ) across all clusters, and the ERS extended into the α/μ band for the prefrontal area. γ band activity was varied by cluster, with PF, AC, and LPM areas showing a decrease in activity compared to quiet standing while LM, RM, and PP areas showed similar or increased synchrony in the γ band. Spectral patterns were generally maintained between passive (Figure 5D) and active (Figure 5E) walking, though μ and β ERD was enhanced during active walking for AC, LM, RM, and PP areas. ERS in the γ band was increased for active walking in the LM, RM, and PP regions.

**Figure 6:**
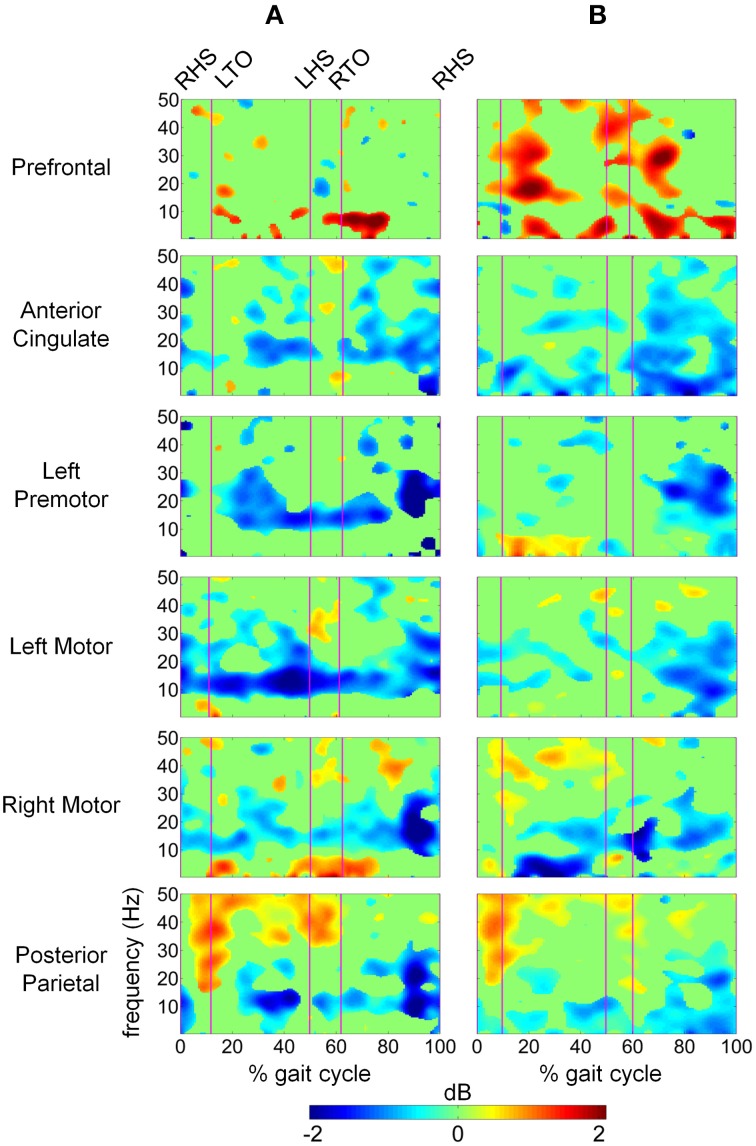
**Time-frequency spectrograms showing the power differences between active and passive walking for (A) slow speed and (B) fast speed in dB**. Spectrograms are masked for significance (α < 0.05) with non-significant differences set to 0 dB (green).

We computed difference spectrograms between active and passive walking for both slow and fast speeds (Figure 6). The difference spectrograms for each speed condition were masked using surrogate statistics with non-significant values set to 0 dB in Figure 6. ERD in the μ band was significantly greater during active treadmill walking in the AC, LPM, LM, RM, and PP regions for most of the gait cycle at both slow and fast speeds, though the enhanced μ ERD was more sustained at the slow speed. Less sustained but still significant β band ERD was observed across these 6 areas as well. In the PF region, significantly increased power was observed in the Δ, θ, and α/μ bands at both slow and fast speeds, while ERS for the upper β and lower γ bands was mostly restricted to walking at the fast pace (Figure 6B). An increase in γ band synchrony was also observed in the PP region, particularly during double stance, while increases in the PF region were strongest during early swing phase. Sporadic γ ERS was also observed in the RM and LM regions during active compared to passive walking.

## Discussion

We observed significantly less power—as indicated by area under the PSD curve (Figure 4) and sustained desynchronization (Figure 6)—in the μ band of LM, RM, and PP regions during active compared to passive treadmill walking. Active walking also resulted in a significant decrease in the β band PSD in the RM and PP areas. Desynchronization in these sensorimotor areas indicates a departure from baseline oscillatory activity and is therefore considered an electrophysiological correlate of increased cortical activation for movement production and/or processing of sensory information (Pfurtscheller and Lopes da Silva, 1999). Therefore, our results indicate that walking on the active treadmill enhanced cortical involvement in the gait task. We also observed significant speed related desynchronization, i.e., less power at faster pace, in the μ band of the LM and PP cluster and β band of PP, suggesting that increased gait velocity also enhanced cortical involvement in the walking task.

Participants were asked to track slow and fast target speeds in both passive and active walking modes; in the passive mode, treadmill belt speed was externally set and maintained at the specified target speed, making the tracking task trivial. In active mode, intentional changes in the swing foot velocity and pelvis position of the participant caused the belt speed to react in real time, resulting in a more challenging tracking task. Data analysis focused only on periods of steady state tracking at slow and fast speeds. Our behavioral data reflect the difficulty disparity between treadmill modes as we observed more step-to-step variability in belt speed during active walking for both speeds (Figure 3A). However, the belt speed variability did not degrade overall task performance as no statistically significant differences in pelvis velocity were observed between the two treadmill modes (Figure 3B).

Previous work (Gwin et al., 2011; Severens et al., 2012 Wagner et al., 2012, 2014; Seeber et al., 2014) has demonstrated that cortical activity is modulated relative to the gait-cycle. In agreement with these findings, we observed gait-cycle related modulations of μ, β, and γ power in the left motor, right motor, and posterior parietal cortices (Figures 5B,C). Interestingly, gait phase modulated activity was enhanced in the left premotor and anterior cingulate regions during active treadmill walking, while there was little difference between active and passive walking in other areas of the cortex. We also observed sustained μ (8–13 Hz) and β (14–30 Hz) desynchronization in the left premotor, left motor, right motor, and posterior parietal cortices during walking compared to rest (Figures 5D,E). When comparing active to passive treadmill walking, there was consistent μ band desynchronization in all clusters except prefrontal cortex (Figure 6). β band ERD for active compared to passive treadmill was also present in these same clusters, although less prominently than in the μ band. Numerous studies have correlated desynchronization in these frequency bands with activation of the sensorimotor system (Pfurtscheller and Lopes da Silva, 1999; Miller et al., 2007) and in particular with involvement of sensorimotor cortices in precision stepping, robotic assisted stepping, and walking (Wieser et al., 2010; Presacco et al., 2011; Wagner et al., 2012). We believe desynchronization in these bands represents enhanced cortical attention to the motor tasks during active walking, an observation supported by the more variable step-to-step belt velocity during the active condition (Figure 3A). Mean pelvis velocity was similar between the active and passive conditions, thus these results demonstrate that during active treadmill walking participants were more attentive to the task. Our results are in agreement with recent studies which found sustained μ and β ERD in the sensorimotor and parietal cortices of healthy individuals while they actively walked with a robotic gait trainer (Wagner et al., 2012; Seeber et al., 2014). The authors of the second study posited that the ERD reflected a movement related active neuronal state present throughout the gait cycle. The increased ERD we observed during simulated overground walking while tracking a target speed further bolsters this active neuronal state hypothesis. Our results also align with previous studies showing increased activity in sensorimotor and premotor areas during a wide variety of active compared to passive movements, including ankle dorsiflexion (Dobkin et al., 2004), pedaling (Hollnagel et al., 2011; Jain et al., 2013), foot movement (Muller-Putz et al., 2007), and walking with a robotic gait trainer (Wagner et al., 2012). A similar increase in ERD was observed between balance beam and normal treadmill walking (Sipp et al., 2013). We also observed sporadic—but significant—increases in theta band power for active vs. passive walking in the PF, LPM, and RM clusters at varying points in the gait cycle, as well as increased θ band PSD in the PF cluster during fast walking. This increased θ band activity may be related to correction of error between target and current treadmill speed, a result consistent with previous EEG studies demonstrating θ band response in the anterior cingulate and sensorimotor areas in response to motor or performance errors (Slobounov et al., 2009; Sipp et al., 2013). Our results also support the theory of a shared control paradigm for locomotion, in which the rhythmic movements themselves are generated by spinal networks but can be modulated by supraspinal structures (Rossignol et al., 2006).

Interestingly, power in the low γ band in prefrontal, sensorimotor, and posterior parietal regions was increased when comparing active to passive treadmill walking adding to the emerging body of evidence implicating these oscillations in coordination and control of peripheral movement. Other studies of have shown that low γ band oscillations are dynamically modulated relative to gait cycle phase (Gwin et al., 2011; Wagner et al., 2012, 2014; Seeber et al., 2014). Such gait-related modulations could be task related; peak corticomuscular coherence appears to shift to low γ band during isotonic lower extremity movements (Gwin and Ferris, 2012) and walking (Petersen et al., 2012). Low γ oscillations in the sensorimotor area were altered when participants attempted to walk with a robotic gait trainer compared to when the robot passively moved their legs (Wagner et al., 2012) and when the virtual environment in which participants walked was changed (Wagner et al., 2014). Our results demonstrate that low γ band synchronization is enhanced in sensorimotor and posterior parietal cortex while participants tracked a target speed using the active treadmill. This result provides additional evidence for the role of cortical circuits in controlling human locomotion. It also supports the hypothesis that the sensorimotor system shifts its oscillatory mode of operation toward higher frequencies in situations demanding dynamic motor control. This theory is supported by previous studies demonstrating an increase in low γ band corticomuscular coherence when participants are required to track a force output profile with their fingers (Omlor et al., 2007; Mehrkanoon et al., 2014); the latter study posited that the γ band activity may play a role in error correction since the increased coherence was correlated with target overshoot. Based on prior studies implicating γ synchronization as a fundamental contributor to cortical computation (Fries, 2009), spatial and visual attention (Rouhinen et al., 2013), and coordination of functional cell assemblies (Canolty et al., 2010) we hypothesize that the operational shift to low γ band enables the sensorimotor system to effectively integrate visual and somatosensory feedback to plan and execute more complex movements. Our results are indicative of such system performance in a dynamic walking task requiring tracking of a target gait speed. In particular, posterior parietal γ band synchronization was significantly increased during double stance and early swing phases of active treadmill walking (Figure 6). The active treadmill controller adjusts belt speed primarily based on peak swing foot velocity, which occurs during early- to mid-swing phase (Yoon et al., 2012); thus, these gait-phase specific bursts in parietal γ band activity may demonstrate increased attention to foot velocity to accurately track the target walking speed.

Active treadmill walking also elicited a broadband increase in prefrontal cortex activity compared to passive treadmill, especially during fast walking (Figure 6). This result is in line with previous fNIRS studies demonstrating increased prefrontal activity during speed adaptation on a treadmill (Suzuki et al., 2004) and precision stepping (Koenraadt et al., 2014). Notably, these studies showed that prefrontal activation was further elevated during faster gait speeds and immediately following the transition to the precision walking task. Our results follow a similar pattern. One reason for this increased activation could be the role of prefrontal cortex in top-down control of behavior, particularly for non-simplistic motor commands requiring novel integration of sensory and cognitive inputs, or when these inputs are rapidly changing (Miller and Cohen, 2001). Recent theory suggests communication from the posterior medial frontal cortex to the lateral prefrontal cortex, which in turn communicates with task-related motor areas, as a mechanism for initiation of this top-down control (Danielmeier et al., 2011). This type of executive function for effective task performance has been linked to gait, and in particular, the top-down control appears to grow stronger as the locomotor task becomes more challenging (Yogev-Seligmann et al., 2008); our results are consistent with this observation. Furthermore, fMRI studies have implicated a dorsal frontoparietal network for control of visuo-spatial attention (Corbetta and Shulman, 2002). In our study, participants tracked target gait speeds while visually receiving real-time time feedback of their performance via a status bar on a screen in front of the treadmill. Our results suggest that the active treadmill enhanced top-down attention to the walking task via elevated activity of this frontoparietal network, perhaps to adjust leg motion to match the target treadmill speed. This visuo-spatial attentional network has been implicated in action selection of different motor effectors, including movement of the arms and hands (Gallivan et al., 2011). Our study presents evidence that this network may play a similar role in control of the lower extremities during a precision walking task.

Similar regions of the cortex are crucial for motor learning and skill acquisition. Indeed, rapid motor learning, the type resulting from a single training session, appears to, at least initially, involve activation of dorsolateral prefrontal cortex, pre-motor, and primary motor areas, while the supplementary motor area, parietal regions, cerebellum and subcortical structures show increased activity as learning progresses (Dayan and Cohen, 2011). One possible framework for this behavior is the involvement of parallel, task-specific circuits responsible for simultaneous learning of spatial and motor coordinates required for movement execution, in which the former is accomplished via fronto-parietal-striatum-cerebellar circuits while the latter is supported by primary motor-sensorimotor-striatum-cerebeller circuitry (Hikosaka et al., 2002). Learning spatial coordinates may proceed more expeditiously at the expense of attentional and executive resources provided by the pre-frontal cortex (Miller and Cohen, 2001). Interestingly, the active treadmill induced prefrontal cortex activity was elevated for the more challenging walking conditions, i.e., during fast walking, a task which requires rapid integration of spatial information with motor execution.

A limitation of the current study was the multiple within-subject conditions evaluated (2 speed conditions and 2 treadmill control types) which lowered the statistical power of tests on the mean PSD of each cluster due to the multiple comparison correction. The mean PSD collapses the time dimension to capture broad oscillatory changes between walking conditions. To limit the number of statistical comparisons, we chose to segment the PSD into broad frequency ranges at the risk of eliminating differences that may have been identified if smaller frequency bins had been employed. Future studies with more trials can empower more detailed analysis of frequency band specific power changes during walking. Yet, the emergence of μ and β band ERD for active relative to passive and for fast relative to slow walking in the motor and parietal cortices reflects enhanced activity during these tasks, an important result as it pertains to rehabilitation because activation of these motor areas plays a key role in neural plasticity for motor learning (Dayan and Cohen, 2011).

Caution should be exercised when interpreting the location of the dipoles obtained from the finite element model which has been shown to have median errors of 5–8 mm (Akalin Acar and Makeig, 2013). In addition to location information, we also included spectral properties when clustering dipoles across subjects into regions of interest, ensuring that only dipoles which were both similarly located and exhibited similar time-frequency responses were grouped together.

We employed a systematic pre-processing strategy to isolate true electrocortical signals from artifact including, to the best of our knowledge, the first application of ASR (Mullen et al., 2013) to EEG recorded during gait. The 500 ms time windows used for ASR were not tied to the gait cycle and channels identified as corrupted exceeded a threshold variance in principle component space. Removal of stride locked electrocortical activity is possible but unlikely because any signal originating from the cortical surface would be present on multiple electrodes by volume conduction. We conservatively applied ASR, as shown in Figure 2, to obtain sphering and weighting matrices from the AMICA algorithm. The actual ICA transformation was applied to non-ASR cleaned data to minimize possible loss of true cortical activity from the ASR process. Increased power spectral density was present in Δ band during fast walking in the anterior cingulate and right motor regions (Figure 4), an effect that may be attributable to residual motion artifact. However, these increases were not statistically significant, and the presence of well known neural correlates pertaining to movement in other frequency ranges (e.g., a peak in μ power in the right motor cluster) suggests that electrocortical activity was present in bands of primary interest. Unlike previous studies demonstrating artifact contamination of EEG data (Castermans et al., 2014) we observed within stride changes in electrocortical activation which were not broadband in nature but were confined to individual frequency bands previously implicated in motor learning and control that are unlikely to be artifact related. In particular, our statistical comparisons of time-frequency spectrograms evaluated changes in cortical activity across the active and passive treadmill at the same speeds thus the kinematics and related motion artifacts were similar. This procedure was designed to eliminate any residual task-related artifacts while elucidating task-related changes in cortical activity. Such an approach may regrettably eliminate some brain activity which is coupled to the gait cycle and therefore limit conclusions regarding the underlying mechanisms that contribute to supraspinal control of locomotion. But it also highlights cortical circuits that are activated to provide attention to the gait velocity tracking task, information which is also valuable for development of neurorehabilitation paradigms.

## Conclusion

While previous work has examined cortical activity during typical treadmill walking or walking in a robotic assisted gait training device, this study utilized a novel experimental paradigm to differentiate neural contributions to precision speed control during simulated overground walking. We observed significant task related differences in cortical activation patterns between active and passive treadmill walking across multiple regions. Specifically, μ and β band desynchronization was enhanced in the sensorimotor areas indicating increased cortical involvement in the active walking task. Phasic γ band synchronization was observed in the prefrontal and posterior parietal areas during simulated overground walking, providing evidence for γ band oscillations in control of bipedal locomotion. Prefrontal synchronizations were enhanced for the fast speed, suggesting executive control of sensorimotor areas is elevated to improve speed tracking performance. It is important to note that engagement of these cortical structures do not, in and of themselves, provide evidence for neuroplasticity or the efficacy of this active treadmill training approach. Careful assessment of functional outcomes via randomized controlled trials comparing these two different types of treadmill control is necessary to correlate these alterations in brain activity with improved therapeutic benefits. Such studies are planned in the future. Additionally, our results demonstrate the feasibility and sensitivity of non-invasive EEG for monitoring brain activity during gait, supporting its future role as a clinical tool for rehabilitation paradigm development and assessment.

### Conflict of interest statement

The authors declare that the research was conducted in the absence of any commercial or financial relationships that could be construed as a potential conflict of interest.
