# Supplementary figures and images for: Prefrontal, posterior parietal and sensorimotor network activity underlying speed control during walking
Source: Front Hum Neurosci. 2015 May 12;9:247. doi: 10.3389/fnhum.2015.00247 (PMC4429238; doi:10.3389/fnhum.2015.00247)

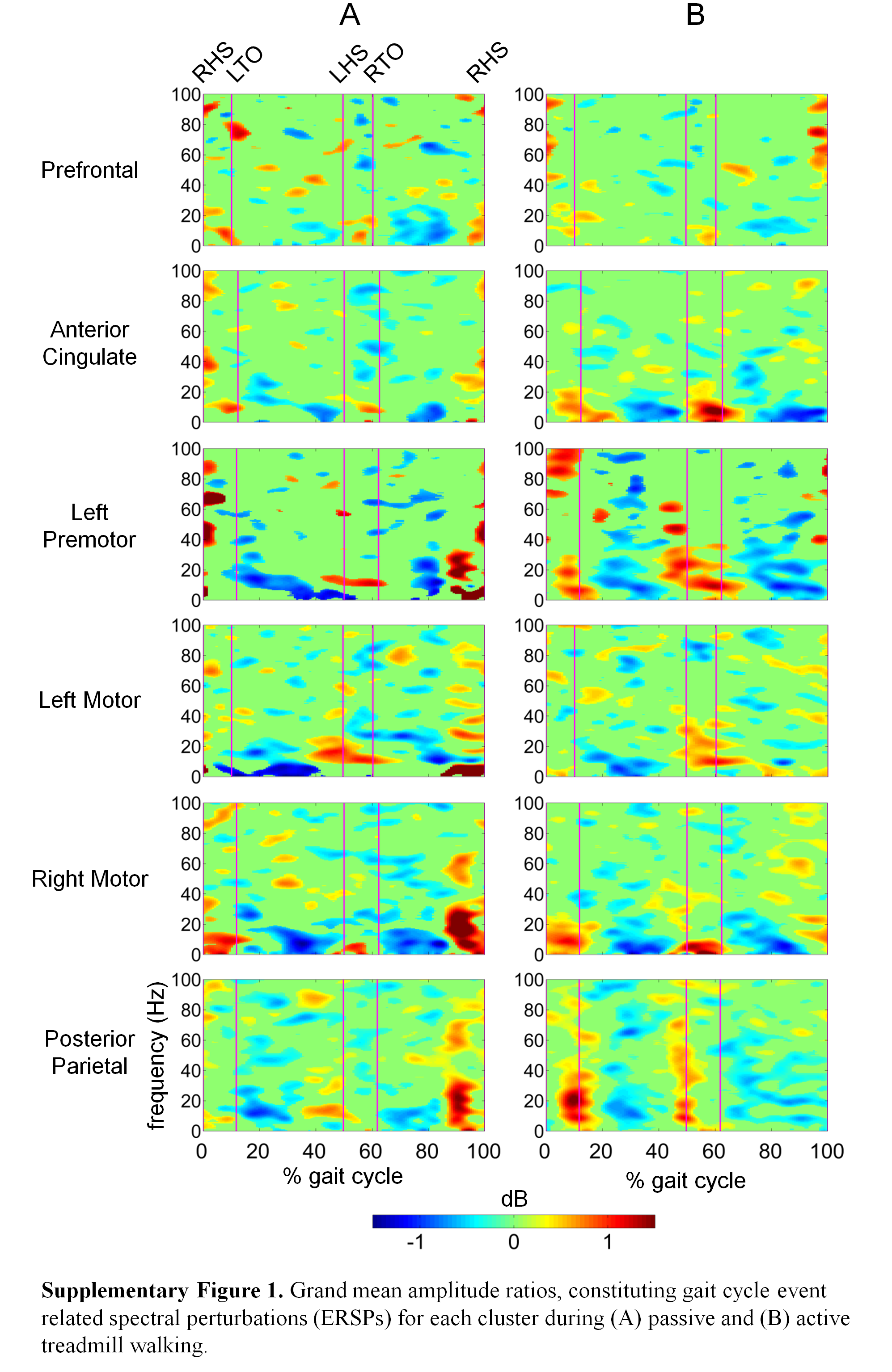

Supplement: Supplementary file 1 [file Image1.TIF]
